# Solution Structure of the SGTA Dimerisation Domain and Investigation of Its Interactions with the Ubiquitin-Like Domains of BAG6 and UBL4A

**DOI:** 10.1371/journal.pone.0113281

**Published:** 2014-11-21

**Authors:** John F. Darby, Ewelina M. Krysztofinska, Peter J. Simpson, Aline C. Simon, Pawel Leznicki, Newran Sriskandarajah, David S. Bishop, Lisa R. Hale, Caterina Alfano, Maria R. Conte, Santiago Martínez-Lumbreras, Arjun Thapaliya, Stephen High, Rivka L. Isaacson

**Affiliations:** 1 Department of Chemistry, King's College London, Britannia House, 7 Trinity Street, London, SE1 1DB, United Kingdom; 2 Division of Molecular Biosciences, Imperial College London, Exhibition Road, South Kensington, London, SW7 2AZ, United Kingdom; 3 Bruker U.K. Ltd, Banner Lane, Coventry CV4 9GH, United Kingdom; 4 Faculty of Life Sciences, University of Manchester, The Michael Smith Building, Oxford Road, Manchester, M13 9PT, United Kingdom; 5 Randall Division of Cell and Molecular Biophysics, King's College London, New Hunt's House, London SE1 1UL, United Kingdom; Università di Napoli Federico II, Italy

## Abstract

***Background*:**

The BAG6 complex resides in the cytosol and acts as a sorting point to target diverse hydrophobic protein substrates along their appropriate paths, including proteasomal degradation and ER membrane insertion. Composed of a trimeric complex of BAG6, TRC35 and UBL4A, the BAG6 complex is closely associated with SGTA, a co-chaperone from which it can obtain hydrophobic substrates.

***Methodology and Principal Findings*:**

SGTA consists of an N-terminal dimerisation domain (SGTA_NT), a central tetratricopeptide repeat (TPR) domain, and a glutamine rich region towards the C-terminus. Here we solve a solution structure of the SGTA dimerisation domain and use biophysical techniques to investigate its interaction with two different UBL domains from the BAG6 complex. The SGTA_NT structure is a dimer with a tight hydrophobic interface connecting two sets of four alpha helices. Using a combination of NMR chemical shift perturbation, isothermal titration calorimetry (ITC) and microscale thermophoresis (MST) experiments we have biochemically characterised the interactions of SGTA with components of the BAG6 complex, the ubiquitin-like domain (UBL) containing proteins UBL4A and BAG6. We demonstrate that the UBL domains from UBL4A and BAG6 directly compete for binding to SGTA at the same site. Using a combination of structural and interaction data we have implemented the HADDOCK protein-protein interaction docking tool to generate models of the SGTA-UBL complexes.

***Significance*:**

This atomic level information contributes to our understanding of the way in which hydrophobic proteins have their fate decided by the collaboration between SGTA and the BAG6 complex.

## Introduction

The BAG6 complex is composed of the proteins BAG6 (BCL2-associated athanogene 6 [1]), TRC35 (transmembrane recognition complex 35 [2]) and UBL4A (ubiquitin-like protein 4A [2]) and, while resident in the cytoplasm, has a key role in several protein quality control pathways through transient binding to numerous other proteins including components of the ribosome [2] and proteasome [3]. It is also involved in the DNA damage response when localised to the cell nucleus via the nuclear localisation signal on BAG6 [4]. Cytoplasmic quality control mechanisms include the endoplasmic reticulum-associated degradation pathway (ERAD), whereby misfolded proteins are ubiquitinated and retrotranslocated out of the ER and into the cytosol to enable their proteasomal degradation [5]. A range of ER and cytoplasmic components are involved in this process, often displaying substrate specificity in their activity, and in several cases the BAG6 complex has been implicated in the efficient removal of certain ERAD substrates, perhaps acting as a ‘holdase’ to maintain retrotranslocated proteins in a soluble form once they enter the cytosol [6]. Likewise, the BAG6 complex can target membrane and secretory proteins that mislocalise to the cytosol for proteasomal degradation via a pathway that is antagonised by SGTA (small, glutamine-rich, tetratricopeptide repeat-containing protein alpha) [7].

Another group of well-defined substrates for the Bag6 complex are tail-anchored (TA) membrane proteins destined for delivery to the ER via the transmembrane domain recognition complex (TRC) pathway [8]. The TRC pathway receives TA-proteins from the BAG6 complex and transports them into the ER membrane through a series of steps involving the downstream proteins TRC40, WRB and CAML.

As the link between these diverse pathways leading to either biosynthesis or degradation, the BAG6 complex appears to act as a triage point. Hence it receives a diverse range of substrates, each characterised by an exposed hydrophobic region, and maintains them in a soluble state until they can be handed off to the next partner in the relevant pathway. Substrates are passed onward to TRC40 for TA-insertion via the TRC pathway or, in the case of misfolded and mislocalised proteins, to the proteasome for degradation. Recent work has demonstrated that the co-chaperone SGTA is an important cofactor for the BAG6 complex [7], [9], [10]. The ability of SGTA to interact with both the BAG6 complex and an overlapping set of hydrophobic substrates allows it to transfer proteins into the BAG6 system for triage while moderating their fate. SGTA is capable of reversing the ubiquitination instigated by the BAG6 complex, thereby rescuing proteins from degradation [7]. SGTA also interacts with chaperones of the Hsp70 and Hsp90 families and may enable the transfer of client proteins between different cytosolic quality control pathways, although the precise mechanism of SGTA-substrate binding is unresolved [11], [12].

SGTA is a 34 kDa protein that forms a homodimer via an N-terminal dimerisation domain. In addition, SGTA contains a central tetratricopeptide repeat (TPR) domain (that can interact with the C-termini of Hsp70 and Hsp90 molecular chaperones as well as viral proteins and hormone receptors) and a glutamine rich region towards the C-terminus [13]. Although all of its roles have yet to be fully elucidated, SGTA looks to be an important protein for health and disease, recently found to be up-regulated in certain cancers (oesophageal [14], liver [15], ovarian [16] and lung [17]). Recent studies of SGTA, and its yeast homologue Sgt2, have demonstrated that SGTA interacts with the BAG6 complex's two ubiquitin-like (UBL) domains via a ubiquitin-like binding domain (UBD).

UBL domains are highly similar to ubiquitin and in some cases can be appended onto protein substrates as single domains in a manner analogous to ubiquitin. This family is referred to as type I UBLs. Where UBLs are present as functional domains in larger proteins these are known as type II UBLs. Many type II UBLs interact with a broad range of ubiquitin binding domains (UBDs) whereas type I UBLs seem to have highly specific binding partners. Ubiquitin, UBLs and UBDs occur throughout the ubiquitin proteasome system and ERAD pathway as key protein-protein interaction sites. The BAG6 complex contains two type II UBLs, one in the UBL4A subunit which does not appear to bind canonical UBDs but does interact with SGTA. The second, at the N-terminal of BAG6 itself, can interact with canonical UBDs but also retains the ability to bind the unusual UBD of SGTA.

Work focusing on the yeast equivalent of the TRC pathway, the guided entry of TA proteins (GET) pathway, has structurally elucidated the UBL/UBD interaction between the yeast SGTA and UBL4A homologues, Sgt2 and Get5 [18]. In yeast the BAG6 complex step is replaced in the GET pathway by a reduced equivalent formed of Get4 and Get5 [19], in place of TRC35 and UBL4A respectively but with no homologue for BAG6. These structures demonstrate that Sgt2 forms an unusual UBL-binding domain (UBD) which spans the homodimerisation interface resulting in one Get5_UBL-binding site per dimer, thereby creating a versatile branched arrangement between the Sgt2 dimer and the heterotetrameric complex comprising dimers of Get5 and Get4.

In this study we solve a solution structure of the full SGTA dimerisation domain and structurally characterise its interaction with the UBL domains from BAG6 and UBL4A. This new structure and biophysical characterisation of the SGTA-UBL interactions is used to compare in greater detail the differences between binding by UBL4A and BAG6 to the SGTA N-terminus in mammalian systems and contributes towards understanding the role of the BAG6 complex as a triage hub in several protein quality control pathways.

## Results

### NMR solution structure of the SGTA dimerisation domain

**Figure 1:**
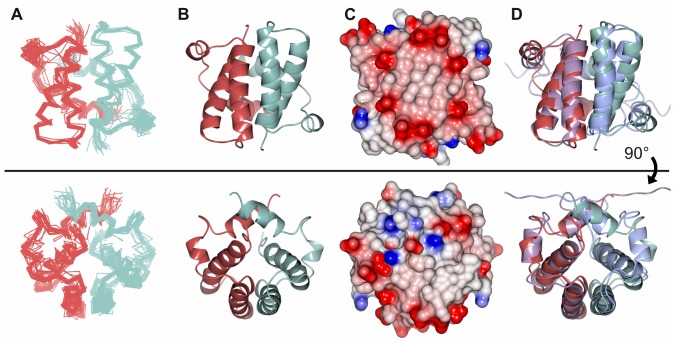
NMR structures of SGTA_NT dimer rotated 90° around the x-axis. A) Ensemble views showing top 20 lowest energy ARIA-calculated structures as deposited in the PDB (Accession code: 4CPG); monomers represented in pale red and sea green B) Ribbon representation with monomers coloured as in A C) Electrostatic views ranging from −10 negative charge in red to +10 positive charge in blue modelled using ccp4mg [37] which calculates the charge distribution displayed on the solvent accessible surface of the protein D) Structural alignment of SGTA_NT (pale red/sea green) with SGT2_NT (lilac/blue; [18]; PDB: 4ASV) superposed using secondary-structure matching in ccp4mg [37]. The structures align with RMSD  = 2.41 Å.

After construct optimisation we were able to complete backbone and sidechain NMR assignments (BMRB Accession Number: 19779) for SGTA_NT, residues 1–69. We proceeded to solve the solution structure of this domain by NMR spectroscopy using standard methods and mixed isotope labelling, analogous to our previous approach [18]. Like its yeast counterpart, SGTA_NT forms a tight symmetrical homodimer (Figure 1; PDB Accession Number: 4CPG; Structural statistics in Table S1). Each monomer consists of four alpha helices (α1 =  N3-H21; α2 =  S26-F43; α3 =  V47-L52; α4 =  L58-T67) connected by short loops and arranged in a fold analogous to that of Sgt2_NT. The dimer interface is highly hydrophobic resembling the core of a globular protein and spans an area of 1266 Å^2^ as calculated by PISA [20]. A structural overlay with the equivalent yeast domain is shown in Figure 1D.

### *Binding interface with* UBL4A_UBL domain

**Figure 2:**
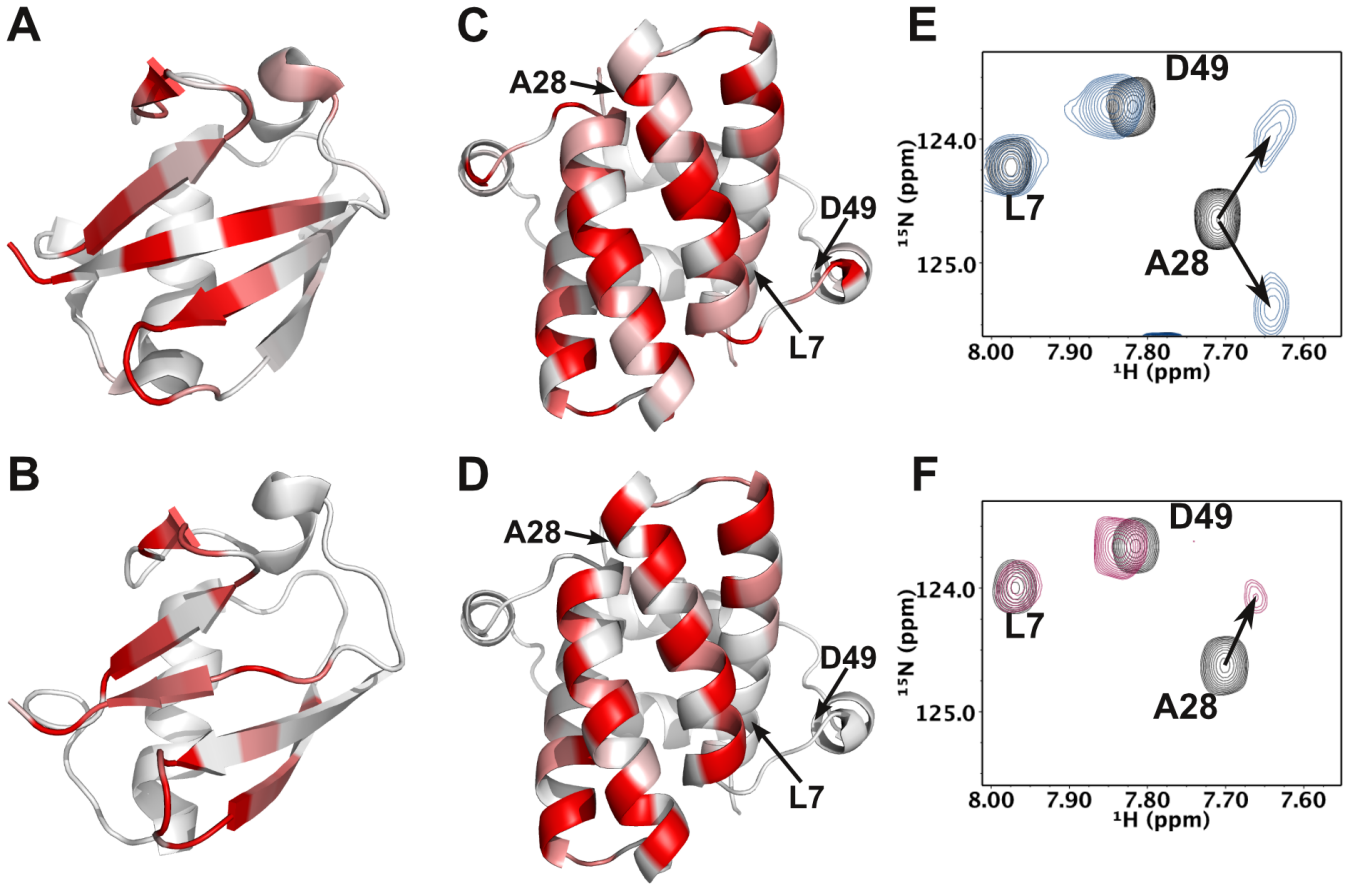
Chemical shift perturbation data for SGTA_NT/UBL interactions. A–D: Ribbon views coloured according to reciprocal chemical shift perturbation upon binding partner proteins. Residues whose shifts are greater than one standard deviation above the mean chemical shift are coloured darkest red. Those below the mean are coloured white and shifts between these points are graded pink. A) BAG6_UBL B) UBL4A_UBL C) SGTA_NT upon binding BAG6_UBL D) SGTA_NT upon binding UBL4A_UBL; E–F: Region of ^1^H-^15^N HSQC spectra of ^15^N-labelled SGTA_NT before (black) and after (blue/maroon) titration with saturating quantities of unlabelled BAG_UBL (E) and UBL4A_UBL (F) Residue A28 splits upon binding to BAG6_UBL but not upon binding UBL4A_UBL. See Figure S1 for full HSQC data on all split peaks.

The solution structure of the UBL domain from UBL4A has been solved previously by Zhao and colleagues as part of the RIKEN Structural Genomics Initiative and deposited in the Protein Data Bank (Accession Number: 2DZI) with no associated publication to date. Since the NMR chemical shifts were not originally made public we reassigned the backbone (see Figure S3) for the purpose of interaction experiments [21]. Reciprocal chemical shift perturbation studies carried out by titrating unlabelled UBL4A_UBL into ^15^N-labelled SGTA_NT and vice versa revealed the binding interface between the two domains (Figure 2) which is reminiscent of the equivalent complex in yeast [18], albeit with some differences which are discussed below. Also, in common with the yeast complex, only one set of NMR signals from the two domains of the SGTA homodimer is observed in the bound state, despite the expectation that a single UBL domain, binding at the homodimer interface should break the chemical equivalence of some NMR signals in the two domains, resulting in two sets of signals. In the Sgt2_NT/Get5_UBL complex, we, and others [22] found that exchange of the UBL between the two equivalent binding sites on the Sgt2 dimer faster than the NMR chemical shift timescale caused averaging of the frequencies at the elevated temperatures required for analysis (310 K) and we attribute the same observation in the mammalian complex to this effect. The 1∶1 (SGTA dimer: UBL monomer) stoichiometry was confirmed by ITC and MST (see later section).

### Binding interface with BAG6_UBL

The solution structure of the UBL domain from BAG6 was also solved previously by Zhao and colleagues and deposited in the Protein Data Bank (Accession Number: 1WX9) and the BMRB (Accession Number: 11263). Moreover, a crystal structure of this domain was recently deposited by Kozlov *et al*. (PDB Accession Number: 4EEW). Neither of these structures is yet associated with a publication. We therefore used the published BMRB assignments in reciprocal chemical shift perturbation studies carried out by titrating unlabelled BAG6_UBL into ^15^N-labelled SGTA_NT and vice versa. Thus the binding interface between the two domains (Figure 2) was solved. As can be observed by comparing the shift perturbations highlighted in Figure 2 and S2, SGTA_NT binds the BAG6 and UBL4A UBLs at the same site. Whilst essentially the same pattern of shift changes occurs in both cases, the BAG6_UBL binding surface appears slightly less extensive spanning 1–2 fewer amino acids at the N-terminal of helix 2 which is central to the interaction. Furthermore, in contrast to UBL4A_UBL binding, we do observe peak splitting for several residues, as indicated in Figure 2E and S1. This suggests that exchange between the two symmetrically-related binding sites on the SGTA_NT dimer is significantly slower than the NMR chemical shift timescale for BAG6_UBL. The residues whose signals split are H21, G22, A28, A35, Q37 and A42, located in helix 2 and the loop connecting helices 1 and 2. This potentially represents an interesting, albeit subtle, difference in the dynamics inherent in the SGTA/UBL complexes.

### SGTA/UBL complexes

**Figure 3:**
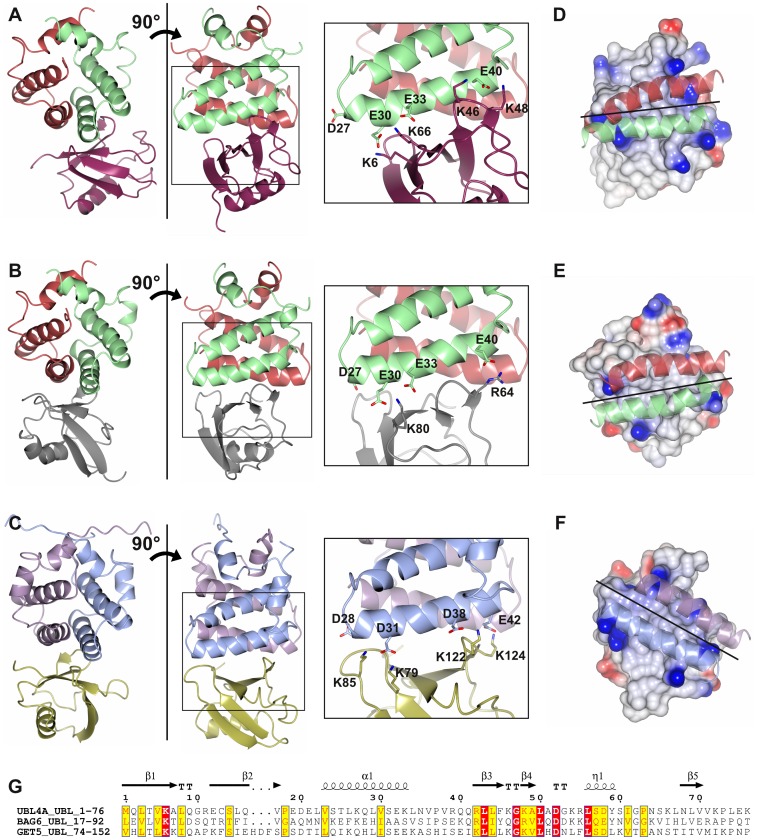
Cartoon representation of the lowest energy complexes of A) SGTA_NT/UBL4A_UBL, B) SGTA_NT/BAG6_UBL and C) Sgt2_NT/Get5_UBL [18] as calculated by HADDOCK from chemical shift perturbation data and intermolecular NOEs. SGTA and Sgt2 are coloured as in Figure 1, the UBLs UBL4A, BAG6 and GET5 are coloured purple, grey and gold respectively. The complexes are aligned by the SGT domain and zoomed-in boxes highlight specific residues involved in each interaction at one side of the SGT dimer. D, E and F show space-fill versions of the equivalent aligned UBL domains; surfaces are coloured according to electrostatic charge as in Figure 1C, exhibiting the positively charged residues that mediate the interaction. The binding helices from the relevant SGT proteins are superposed to show the relative orientations of binding. G) Sequence alignment between the three UBL domains – boxes indicated conserved residues while red highlights sequence identity, structural motifs are labelled across the top with ‘TT’ indicating a β turn. Sequences are numbered according to the UBL4A sequence. Graphic produced using ESPript 3.0 server [38].

Although the structures of SGTA_NT and the UBL domains studied here are highly reminiscent of the yeast equivalent studied previously, the chemical shift mapping data may indicate some subtle differences. Specifically, groups of residues at the start of strands β3 and β5 (positions 39–42 and 66–68 in the sequence alignment shown in Figure 3G) in the UBL domains showed large perturbations in the mammalian but not the yeast system. The binding site clearly occupies the same surface but there are differences in the locations of charged residues known to be important for the Sgt2/Get5 interaction from mutagenesis data by our group and others. Hence we sought to investigate this structurally by generating models of the SGTA_NT/UBL4A_UBL (Figures 3A and S5) and SGTA_NT/BAG6_UBL (Figures 3B and S4) complexes using HADDOCK-based semi-rigid, data-driven docking, an approach we successfully applied to the Sgt2/Get5_UBL complex previously [18]. To supplement the chemical shift perturbation data we ran filtered 3D NOESY experiments on samples comprising unlabeled SGTA_NT and ^15^N, ^13^C-UBL4A_UBL or ^15^N, ^13^C-BAG6_UBL. These generated additional ambiguous restraints which were used in our HADDOCK calculations.

As highlighted in Figure 3 the orientation of the SGTA binding helices relative to the UBL partner in the mammalian complexes is rotated by approximately 45 degrees with respect to the yeast complex. Since the interaction is electrostatically mediated this reflects the differences in negative charge distribution on the central binding helices of SGTA_NT and Sgt2_NT and the differences in positive charge distribution on the surface of the UBLs (details shown in Figure 3). Lys85 in the yeast system's Get5_UBL aligns to Glu12 in human UBL4A_UBL and hence cannot form the same salt bridge. Instead UBL4A_UBL Lys66 and Lys46 pull the SGTA_NT domain around slightly to satisfy the charge interactions. From the SGT perspective, the four negatively-charged amino acids are distributed along the helix differently in Sgt2 and SGTA, with Sgt2 displaying two at each end (D28/D31 and D38/E42) and SGTA showing three at one end (D27/E30/E33 where the Lys85 interaction was lost) and one at the other (E40). The variations in charge distribution of key interacting residues explain the moderate differences in binding mode that occur in the mammalian system.

### BAG6_UBL and UBL4A_UBL compete for the same binding site on SGTA_NT

**Figure 4:**
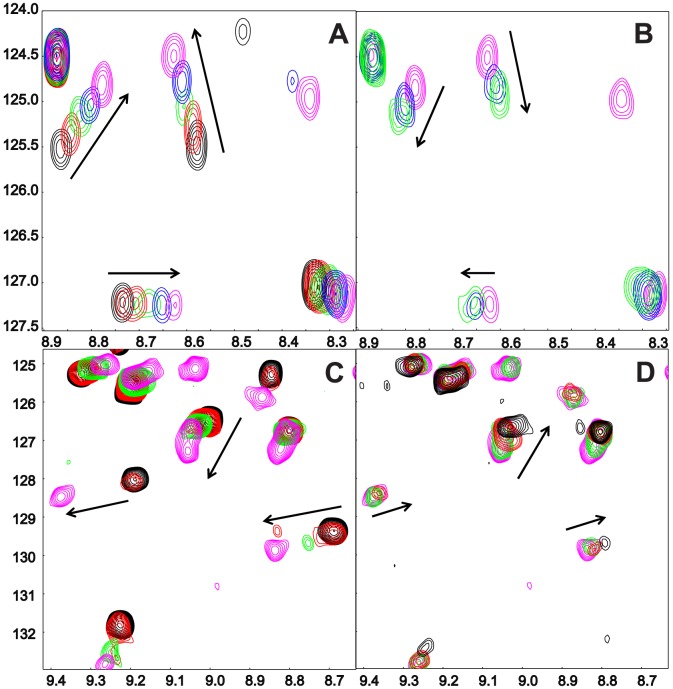
Competitive binding experiments. Region of ^1^H-^15^N HSQC spectra of A) ^15^N-labelled UBL4A_UBL before (black) and after (red/green/blue/magenta) titration with increasing quantities of unlabelled SGTA_NT; B) Endpoint of A with binding competed out through addition of unlabelled BAG6_UBL; C) ^15^N-labelled BAG6_UBL before (black) and after (red/green/magenta) titration with increasing quantities of unlabelled SGTA_NT; B) Endpoint of C with binding competed out through addition of unlabelled UBL4A_UBL. In B and D the bound UBL peaks move back towards their unbound state as their unlabelled equivalents sequester the SGTA_NT.

Given the relationship we observed between the two different UBLs binding to SGTA_NT we wondered whether they bound competitively or if it might even be possible for both to bind simultaneously. To test this we titrated unlabelled SGTA_NT into ^15^N-labelled BAG6_UBL up to a ratio of 2∶1 and monitored the characteristic NMR chemical shift changes that occurred upon binding (Figure 4C). We then added unlabelled UBL4A_UBL and saw the BAG6_UBL peaks shift back towards their unbound state as the availability of SGTA_NT decreased (Figure 4D). We also performed the reciprocal experiment in which ^15^N-labelled UBL4A_UBL was used as the starting point and then titrated with unlabelled SGTA_NT (Figure 4A) followed by unlabelled BAG6_UBL (Figure 4B). Thus, in excess, the UBLs displace each other from SGTA, indicating that they both compete for the same binding site and both species cannot be accommodated simultaneously. Under the conditions used, both UBLs appear to bind with comparable affinity as observed by monitoring the 1D spectra, although we cannot rule out the possibility that *in vivo* the relative affinities are tailored by additional cofactors or other domains of BAG6 or UBL4A.

### ITC and MST

**Figure 5:**
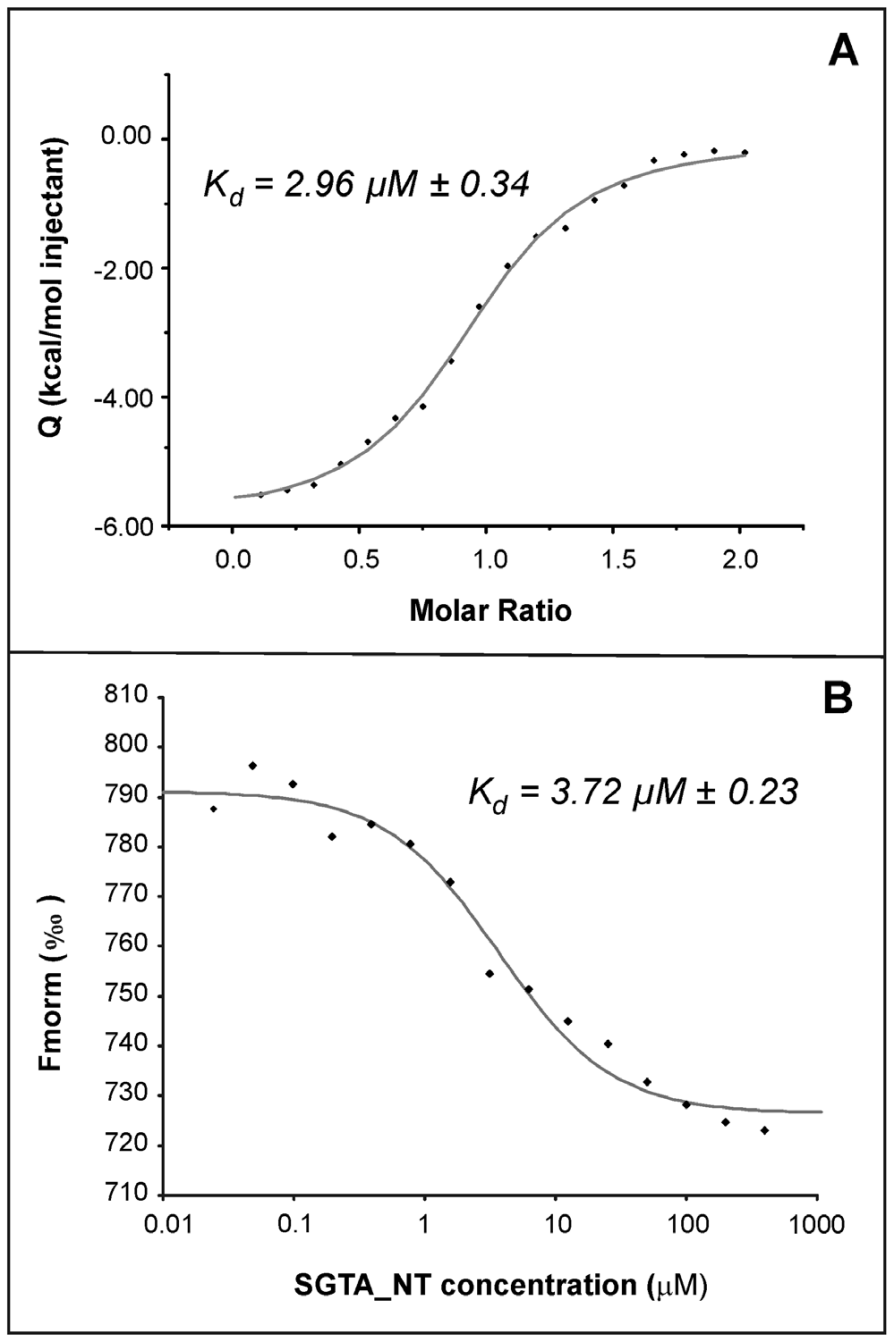
Data showing binding of one UBL4A_UBL domain per dimer of SGTA_NT as determined by A) ITC and B) MST; Dissociation constants (K_d_) are shown for each interaction. In A) The normalized heat of interaction for the titrations was obtained by integrating the raw data and subtracting the heat of ligand (dimer) dilution into the buffer alone. The grey line represents the best fit obtained by a non-linear least squares procedure based on an independent binding sites model.

To examine the stoichiometry of the SGTA_NT/UBL4A_UBL complex empirically, we carried out Isothermal Titration Calorimetry and established that, just like in the equivalent yeast system, one UBL4A_UBL monomer interacts with each SGTA_NT dimer (N = 0.949±0.013; ΔH  = 5900±110; Figure 5A) albeit with a dissociation constant (K_d_) of 3.0 µM, an order of magnitude weaker than in the yeast system. In addition we measured these parameters independently using microscale thermophoresis (MST) which monitors changes in hydration shell, charge and size of molecules across a laser-induced temperature gradient [23]. We arrived at a comparable K_d_ value of 3.7 µM for the binding between SGTA_NT and UBL4A_UBL (Figure 5B). Despite many and varied experimental efforts we were unable to obtain comparably informative data for the interaction between BAG6_UBL and SGTA_NT (see supplemental section for a summary of our approaches and results). ITC experiments confirmed binding between SGTA_NT and BAG6_UBL, whose association generated a well-interpolated, sigmoid-shaped curve based on an independent and equivalent binding sites model. However, the value of N deviated from 1 and an accurate K_d_ could not be determined as these data did not fit any model with viable stoichiometry. We were unable to see any interaction at all using MST. This is despite the NMR chemical shift mapping and filtered NOESY data which unequivocally indicate an interaction between SGTA_NT and BAG6_UBL and the competition titrations which qualitatively imply a similar binding affinity for the two UBLs. The interaction between SGTA_NT and BAG6_UBL is also clearly observable by size exclusion chromatography as demonstrated in Figure S7.

## Discussion

The very recent past saw a number of simultaneous publications from different research groups providing new insights into SGTA, its relationship with the BAG6 holdase complex and the equivalent yeast system including [10], [18], [22], [24], [25]. As a result of the concurrent timing most of these studies did not have the benefit of knowing about each other during their preparation and hence there is some overlap. Here we seek to consolidate the new information in the context of our latest molecular-level contributions to the field as presented here.

### SGTA_NT dimer in structural context

SGTA and its yeast counterpart, Sgt2, share 32% sequence identity and a linear three-domain structure comprised of an N-terminal dimerization domain, a central TPR domain and C-terminal glutamine-rich domain. Until recently the only structural information solved for these proteins was a crystal structure of the TPR domain from SGTA [13]. Our group solved the solution structure of the Sgt2 dimerisation domain (Sgt2_NT) which forms a novel helical fold [18]. Various other Sgt2/SGTA structures have also been added to the literature, namely NMR solution [22] and X-ray crystal [25] structures of partial forms of Sgt2_NT in the context of its complex with Get5, and SGTA_NT [22] encompassing the first two or three out of the four helices in each monomer that we present here. These structures largely agree with each other, differing predominantly in the C-terminal regions remote from the UBL binding interface, apparently as a result of the use of truncated constructs of SGTA and Sgt2 in the latter studies. In our initial construct optimisation we found that very subtle changes to the domain boundaries had significant effects on secondary structure elements (detailed in [21]). Our new SGTA_NT structure aligns well with the yeast homologue with an RMSD of 2.4 Å. The minor differences between the structures occur at helix α3 and the loop between helices α2 and α3 (see Figure 1D) which form part of the UBL interaction site. These differences may reflect the ability of SGTA to differentially bind to the UBLs from BAG6 and UBL4A where Sgt2 is only thus far known to bind the UBL from UBL4A homologue, Get5, and has no known equivalent for the BAG6_UBL.

### SGTA_NT binding to UBLs

In contrast to the yeast system where there is a single type of higher-affinity UBL on the Get4/Get5 complex for Sgt2, the larger mammalian BAG6 complex contains at least two distinct UBLs capable of binding to SGTA, each with comparable affinity that is lower than that seen in yeast. Our results also indicate that the mammalian UBLs bind to SGTA in a slightly different orientation to the equivalent yeast interaction. These differences may reflect the seemingly more complex role of SGTA as compared to Sgt2. Current models suggest that SGTA/Sgt2 acts upstream of the BAG6/Get4+Get5 complexes, binding hydrophobic substrates before handing them on to BAG6/Get4+Get5 [12], [22]. However, whilst the primary function of Get4+Get5 appears to be the hand off of TA-proteins to Get3 for ER delivery, the BAG6 complex can direct substrates towards distinct outcomes and we speculate that this may be reflected in its more complicated structure. Hence, it is possible that one of the factors that influences the fate of a hydrophobic substrate bound to SGTA is which of the two UBLs located on the BAG6 complex it interacts with prior to substrate release. Thus, one of the UBLs might favour substrate transfer to TRC40 for ER delivery, as observed for TA-proteins [2], [26], whilst the other UBL may promote the BAG6-facilitated ubiquitination of aberrant substrates including mislocalised membrane proteins [3] and ERAD substrates in the process of being retrotranslocated out of the ER membrane [27], [28]. In reality, the process is likely to be even more complex, since the heterotrimeric BAG6 complex appears to form homo-oligomers [29] that would provide multiple UBL domains to which SGTA and/or other interacting partners might be recruited, whilst the polyubiquitination of UBL4A regulates BAG6 activity via targeted proteolysis [30].

The UBL domains of BAG6 and UBL4A presumably decorate the surface of the BAG6 ‘holdase’, although the exact stoichiometry and composition of this complex has yet to be verified. It seems likely that TRC35 and UBL4A exist as dimers, as do their yeast counterparts, although UBL4A lacks the N-terminal domain of Get5 which forms the main contacts to Get4. Part of BAG6 might serve to bridge this gap in the mammalian complex. At least two copies of BAG6 are thought to contribute to each ‘holdase’ but since BAG6 is so much larger than the other components it is possible that a single SGTA dimer has access to, at best, two UBLs from UBL4A and a single UBL from BAG6 at any one time. If the data we measured on isolated domains reflects their behaviour in the larger biological system then the differential on-off rates between the two different UBLs might be an important feature of the fate-determining mechanism for hydrophobic proteins. Further structural and functional work is necessary to fully elucidate this process.

## Materials and Methods

### Protein production

Gene fragments encoding the N-terminal region (residues 1–69) of SGTA, and the UBL domains of UBL4A (1–74) and BAG6 (1–101) were PCR amplified and inserted via ligation-independent Ek/LIC cloning into pET-46 vector under the control of the T7 promoter. (Primers: SGTA forward: 5′ GACGACAAGATGGACAACAAGAAGCGCCTGGCC 3'; reverse 5' GAGGAGAAGCCCGGTTACTTGCCCGTGGCAGCCGC 3'; UBL4A forward: 5' GACGACGACAAGATGCAGCTGACGGTGAAGGCG 3'; reverse: 5' GAGGAGAAGCCCGGTTACAGGGGTTTGACCACTAGGTT G 3'; BAG6 forward: 5' GACGACGACAAGATGGAACCGAATGATAGTACC 3'; reverse: 5' GAGGAGAAGCCCGGTTAACCAGAGCTTGCACCGCT 3').

SGTA_UBL and UBL4A_UBL were transformed into Rosetta cells, while BAG6_UBL was transformed into BL21 (DE3) strain. All proteins were induced with 0.3 mM IPTG at OD_600_ = 0.8 and expressed overnight at 18°C. ^15^N-, ^15^N/^13^C-, and ^2^H/^15^N/^13^C-labelled protein samples were prepared according to unlabelled protocols but in M9-based minimal media using correspondingly labelled ammonium chloride (>98% ^15^N), glucose (>99% U-^13^C), and deuterium oxide (>99.9% ^2^H, Sigma Aldrich). Cells were lysed by sonication and protein was purified by affinity chromatography using HisPur Cobalt Resin (Thermo Scientific).

### NMR spectroscopy

All samples were buffer-exchanged by dilution/re-concentration into 100 mM pH 6.0 MES buffer with 150 mM KCl. NMR experiments were performed on samples of >200 µM uniformly ^15^N, ^13^C-labelled protein in either 5 mm (Sigma-Aldrich) Shigemi or standard 5 mm NMR tubes at 35 or 30°C for SGTA and BAG6/UBL4A samples respectively. Structure solution was carried out in a standard fashion as we have previously described in [18], [21]. Specifically, assignments for the SGTA protein backbone were carried out with standard experiments – HNCO, HN(CA)CO, CBCA(CO)NH, and HNCACB. Assignments for side-chain resonances were performed from a combination of HBHA(CBCACO)NH, (H)CCH-TOCSY, H(C)CH-TOCSY, and amide detected H(CCCO)NH-TOCSY and (H)C(CCO)NH-TOCSY experiments. NOE distance restraints and assignment of aromatics rings were derived from 3D, ^13^C-NOESY-HMQC, 3D, ^15^N-NOESY-HSQC and 3D, ^13^C-separated, (^13^C, ^15^N-*F1*)-reject filtered NOESY experiments.

Data were mostly collected on 600 MHz Bruker Avance lll and 800 MHZ Bruker Avance II spectrometers with TCI and TXI cryoprobes respectively, controlled by Topspin3 (Bruker Biospin Ltd). The ^15^N-filtered NOESY was collected on a Bruker 700 MHz Avance spectrometer with a cryoprobe at Kings College London. The 3D, ^15^N-HSQC-NOESY was collected on a homebuilt 950 MHz spectrometer equipped with triple-resonance, triple-axis gradient probehead at the University of Oxford. Data were processed using NMRPipe [31] and analysed in NMRView [32] (One Moon Scientific). Custom-built NMRView modules aided assignment by allowing rapid input for MARS automated assignment and improved handling of sidechain data [33].

### NMR titrations

Samples of SGTA_NT, UBL4A_UBL and BAG6_UBL for titrations were typically 100 µM in 100 mM MES, pH 6.0 with 150 mM KCl. Spectra were recorded in the absence and presence of a binding partner in a suitable range of molar ratios at 30°C. Shift changes were monitored by 1D ^1^H- and 2D ^1^H-^15^N HSQC spectra.

### ITC

ITC experiments were performed at 30°C using an ITC-200 microcalorimeter from Microcal (GE Healthcare) following the standard procedure as reported previously [34]. Proteins were prepared in 100 mM MES, pH 6.0, 200 mM KCl. In each titration, 20 injections of 2 µL each of SGTA_NT (dimer), at a concentration of 500 µM, were added to a sample of BAG6_UBL or UBL4A_UBL respectively at 50 µM (monomer). Integrated heat data obtained for the titrations corrected for heats of dilution were fitted using a nonlinear least-squares minimization algorithm to a theoretical titration curve, using the MicroCal-Origin 7.0 software package. ΔH (reaction enthalpy change in Kcal/mol), K_b_ (equilibrium binding constant in per molar), and n (molar ratio between the proteins in the complex) were the fitting parameters. The reaction entropy, ΔS, was calculated using the relationships ΔG  =  −RT lnK_b_ (*R* = 8.314 J/(mol K), T 303 K) and ΔG  =  ΔH−TΔS. Dissociation constants (*K*_d_) are shown for each interaction.

### MST

Microscale thermophoresis protein-protein interaction studies were performed on the Monolith NT.115 (Nanotemper Technologies, Munich, Germany) using fluorescently labelled proteins as described [23], [35]. Purified UBL4A was labelled using Monolith NT protein labeling kit RED-NHS (Amine Reactive) dye (NanoTemper Technologies GmbH). The labelling reaction was performed according to the manufacturer's instructions in 100 mM MES pH 6.0, 150 mM KCl, 0.5 mM TCEP applying a concentration of 20 µM protein (molar dye : protein ratio ≈2∶1) at RT for 30 min. Unreacted dye was removed with the supplied dye removal columns. The concentration of labelled proteins was adjusted to 0.8 µM with 100 mM MES pH 6.0, 150 mM KCl, 0.5 mM TCEP, 0.0% TWEEN20. Solutions of unlabelled SGTA_NT were serially diluted 1∶1 using the same buffer producing SGTA_NT concentrations ranging from 800 µM to 48.8 nM. For thermophoresis, each SGTA_NT dilution was mixed with an equal volume of one of the labelled UBL proteins, yielding a final concentration of 0.4 µM fluorescently labelled protein and final SGTA_NT concentrations ranging from 400 µM to 24.4 nM. After 15 min incubation, about 5 µl of each solution was loaded into Monolith NT Standard Capillaries (NanoTemper Technologies GmbH). Thermophoresis was measured using a Monolith NT.115 instrument (NanoTemper Technologies GmbH) at an ambient temperature of 25°C with 5 s/30 s/5 s laser off/on/off times, respectively. Instrument parameters were adjusted with 10% LED power and 40% IR-laser power. Data from three independently pipetted measurements were analyzed (NT Analysis software version 1.2.101, NanoTemper Technologies) using the signal from Thermophoresis + T-Jump.

### NMR structure determination of SGTA

The solution structure of the SGTA N-terminal dimer was solved using ARIA2.1 in a similar manner to the Sgt2_NT dimer [18]. NOEs appearing in the filtered NOESY experiment were defined as intermolecular and the corresponding NOEs removed from the 3D ^13^C-NOESY-HSQC with the remainder being treated as solely intramolecular NOEs. NOEs restaints from the ^15^N-NOESY-HSQC were treated as ambiguous. The restraints were applied to a single monomer with the second monomer solved using noncrystallographic symmetry restraints.

Further restraints used were dihedral angles estimated by TALOS and hydrogen bonds in regions containing regular secondary structure (helical i to i+4 H-bonds). The ARIA parameters used for the high temperature, first cooling, and second cooling refine steps were 15,000, 20,000, and 35,000 respectively. The dihedral force constants (high temperature, cool 1, and cool 2 stages) were 40, 80, and 300 Kcal/mol respectively. The non-crystallographic symmetry restraints force constant used was 100 Kcal/mol. Packing to ensure retention of monomer proximity was used throughout, with packing force constants of 15, 10, and 5 Kcal/mol for the high temperature, first cooling, and second cooling steps, respectively. Network anchoring with default parameters was used in iterations 0–4. For each ARIA iteration 40 structures were calculated except the final ensemble the comprised 100 structures, from which the lowest energy 20 structures were subjected to water refinement and deposited to the PDB.

### SGTA/UBL complex docking with HADDOCK

The chemical shift perturbation studies defined clear interaction surface areas in the SGTA_NT/UBL4A_UBL and SGTA_NT/BAG6_UBL complexes. These data were used for complex structure calculation using the HADDOCK approach [36]. In addition, data from intermolecular NOE experiments provided additional restraints. For the calculation, the PDB-deposited structures of UBL4A_UBL (2DZI) and BAG6_UBL (4EEW) and the lowest energy NMR structure from the family of SGTA_NT dimer structures were used. Ambiguous Interaction Restraints (AIRs) were implemented according to the standard protocol. Specifically, from the chemical shift perturbations 20 amino acid residues in SGTA_NT (in both titrations) and 26 and 21 in UBL4A_UBL and BAG6_UBL, respectively, were identified to have weighted chemical shift changes greater than the averages of: 0.055 or 0.083 ppm for SGTA_NT and 0.222 and 0.148 ppm for UBL4A_UBL and BAG6_UBL were chosen as suitable cut-off values. After filtering for a relative solvent accessibility greater than 25%, as calculated using the program Naccess, 14 and 8 residues in each SGTA_NT monomer, for the UBL4A and BAG6 titrations respectively, were identified as active. These were 34, 38, 40, 41, 43, 44, 47, 48, 51, 54, 55, 60, 62, and 64 for the UBL4A titration and 39, 45, 47, 51, 54, 55, 58, and 60 for the BAG6 titration. Similarly for UBL4A_UBL 14 residues, namely 15, 16, 17, 18, 19, 21, 23, 49, 54, 55, 56, 73, 75, and 78 were active. Finally for the BAG6_UBL 16 residues, 22, 23, 24, 25, 26, 58, 62, 63, 65, 67, 76, 83, 85, 86, 87 and 88 were identified as active residues.

Filtered NOESY experiments using a 3D ^13^C F1-filtered, F3-edited NOESY-HSQC pulse sequence were carried out on samples comprising unlabeled SGTA_NT and ^15^N, ^13^C-UBL4A_UBL or ^15^N, ^13^C-BAG6_UBL. The ambiguity of the cross peak resonances due to the chemical shift perturbations upon protein binding meant that obtaining explicit distance restraints to guide docking was not possible. Instead, additional AIRs were used as shown in tables S2 and S3.

Solvent exposed residues juxtaposed to the active residues were automatically termed passive residues by the HADDOCK protocol. One thousand initial complex structures were generated by rigid body energy minimization, and the best 200 by total energy were selected for torsion angle dynamics and subsequent Cartesian dynamics in an explicit water solvent. Default scaling for energy terms was applied. Following the standard benchmarked protocol, cluster analysis of the 200 water-refined structures yielded clear ensembles with the lowest HADDOCK score.

## Supporting Information

Figure S1 **Regions of ^1^H-^15^N HSQC spectra of ^15^N-labelled SGTA_NT before (black) and after (blue/maroon) titration with saturating quantities of unlabelled BAG_UBL (left) and UBL4A_UBL (right).** The selected regions highlight amide backbone peaks which show evidence of splitting due to the slower exchange of BAG6_UBL between its two orientations on the dimer of SGTA_NT.
(TIF)

Figure S2 **Full ^1^H-^15^N HSQC spectra of ^15^N-labelled SGTA_NT before (black) and after (maroon/blue) titration with saturating quantities of unlabelled BAG_UBL (bottom) and UBL4A_UBL (top).**
(TIF)

Figure S3 **Full ^1^H-^15^N HSQC spectra of ^15^N-labelled UBL4A_UBL (top) and BAG6_UBL (bottom) before (black) and after (blue/maroon) titration with saturating quantities of unlabelled SGTA_NT.**
(TIF)

Figure S4 **HADDOCK-generated ensembles showing 10 lowest energy structures from the top-scoring cluster for the complex between SGTA_NT and BAG6_UBL aligned according to SGTA (top) and BAG6 (bottom) structures.**
(TIF)

Figure S5 **HADDOCK-generated ensembles showing 10 lowest energy structures from the top-scoring cluster for the complex between SGTA_NT and UBL4A_UBL aligned according to SGTA (top) and UBL4A (bottom) structures.**
(TIF)

Figure S6 **ITC data showing interaction between BAG6_UBL and SGTA_NT domains.**
(TIF)

Figure S7 **Size Exclusion Column data showing interaction between BAG6_UBL and SGTA_NT domains.**
(TIF)

Table S1 **Summary of Sgta_NT dimer solution structural statistics.**
(DOCX)

Table S2 **HADDOCK ambiguous interaction restraints (AIRs) obtained from filtered NOE experiments for the SGTA_NT and UBL4A_UBL complex.**
(DOCX)

Table S3 **HADDOCK ambiguous interaction restraints (AIRs) obtained from filtered NOE experiments for the SGTA_NT and BAG6_UBL complex.**
(DOCX)

File S1 **Supplemental Information.**
(DOCX)
