# Supplementary figures and images for: Solution Structure of the SGTA Dimerisation Domain and Investigation of Its Interactions with the Ubiquitin-Like Domains of BAG6 and UBL4A
Source: PLoS One. 2014 Nov 21;9(11):e113281. doi: 10.1371/journal.pone.0113281 (PMC4240585; doi:10.1371/journal.pone.0113281)

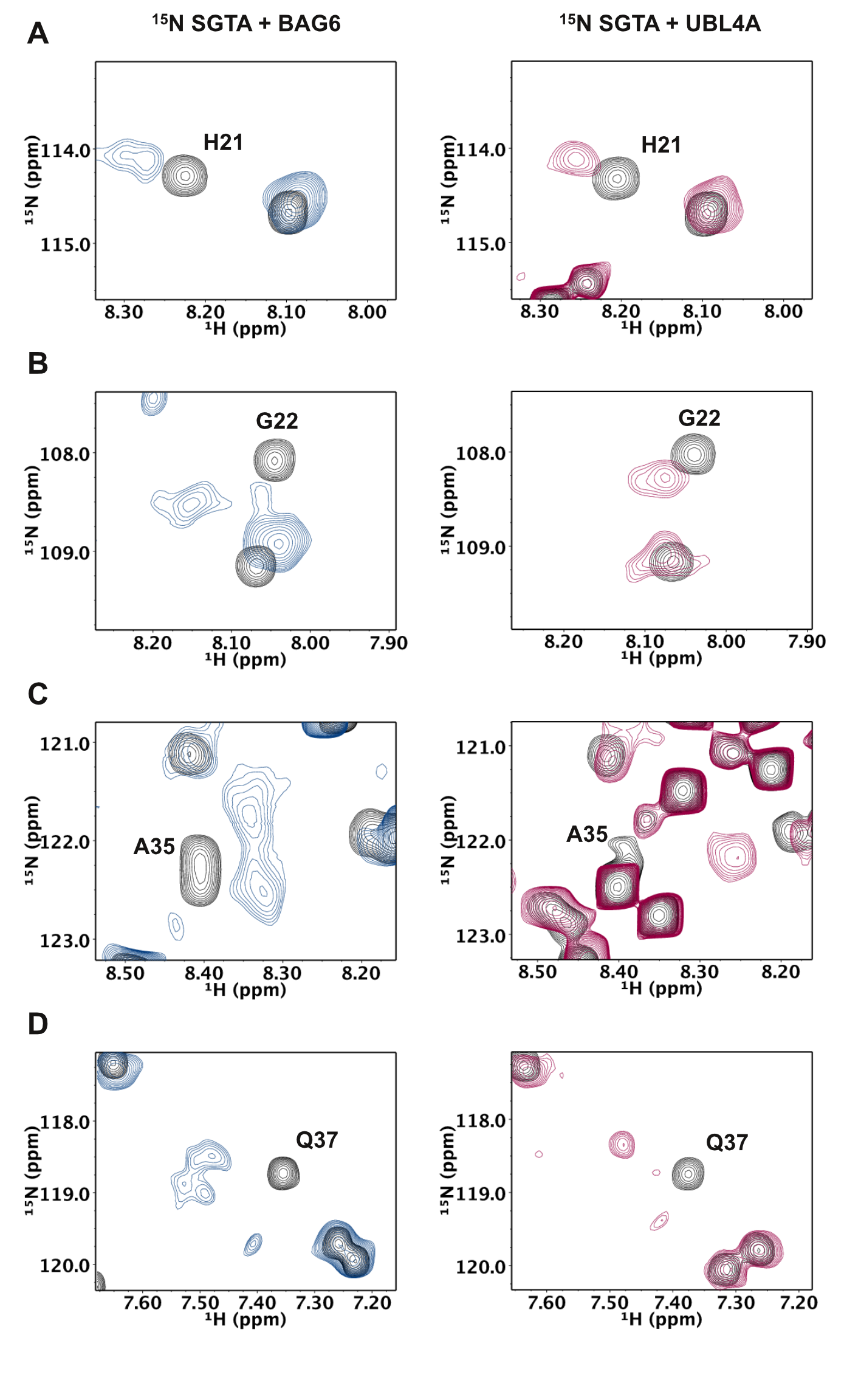

Supplement: Figure S1 — Regions of 1H-15N HSQC spectra of 15N-labelled SGTA_NT before (black) and after (blue/maroon) titration with saturating quantities of unlabelled BAG_UBL (left) and UBL4A_UBL (right). The selected regions highlight amide backbone peaks which show evidence of splitting due to the slower exchange of BAG6_UBL between its two orientations on the dimer of SGTA_NT. (TIF) [file pone.0113281.s001.tif]

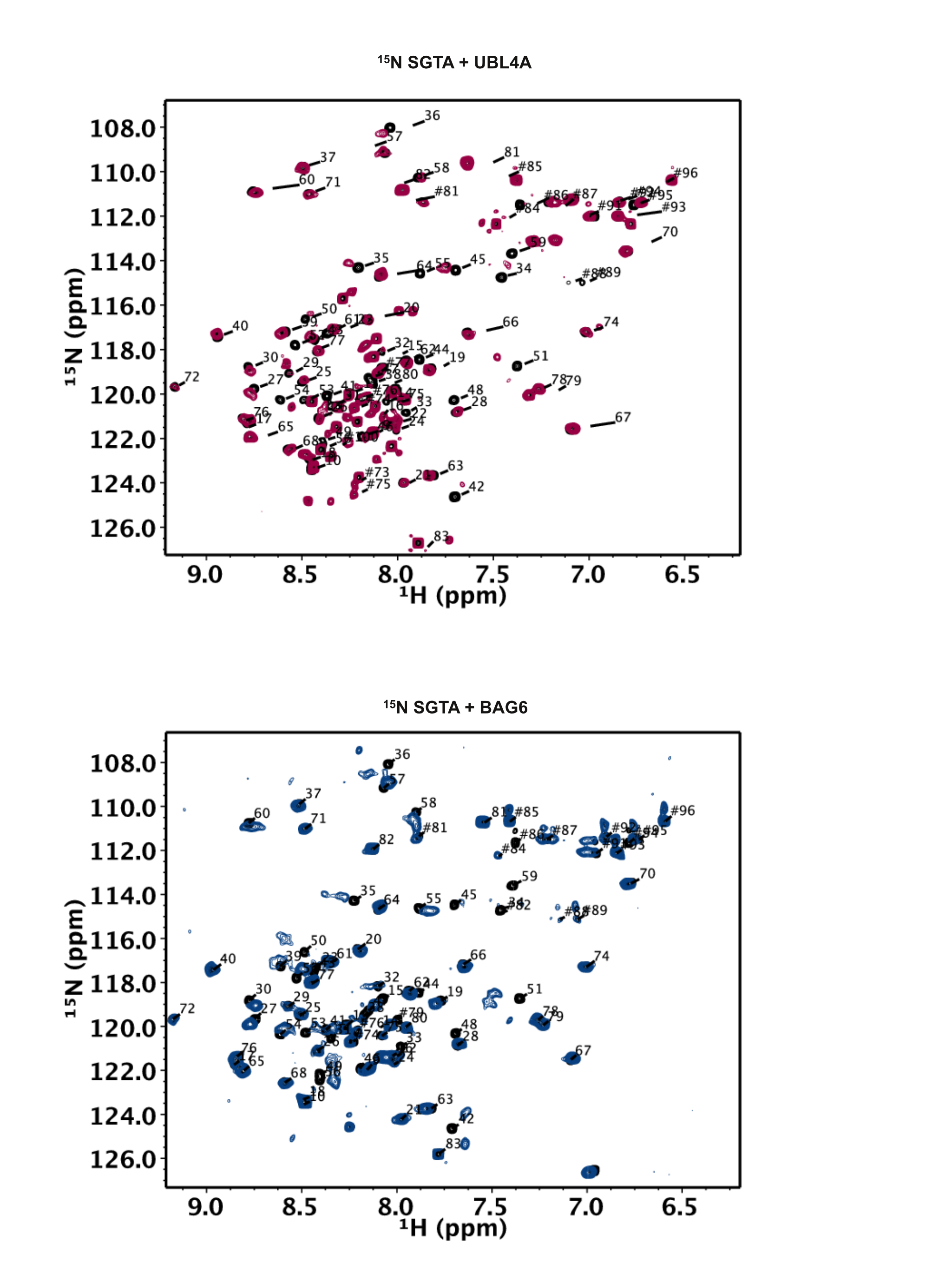

Supplement: Figure S2 — Full 1H-15N HSQC spectra of 15N-labelled SGTA_NT before (black) and after (maroon/blue) titration with saturating quantities of unlabelled BAG_UBL (bottom) and UBL4A_UBL (top). (TIF) [file pone.0113281.s002.tif]

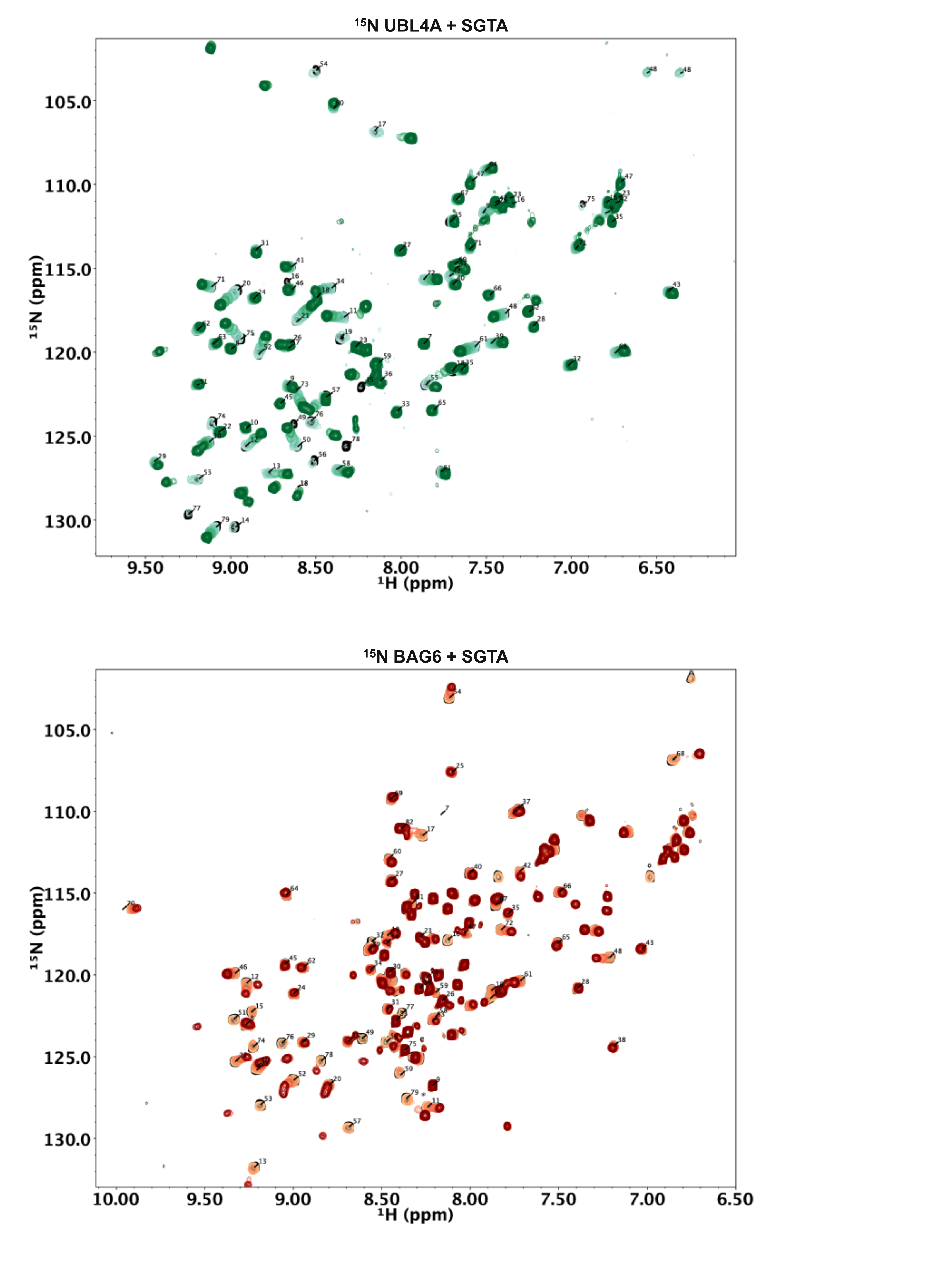

Supplement: Figure S3 — Full 1H-15N HSQC spectra of 15N-labelled UBL4A_UBL (top) and BAG6_UBL (bottom) before (black) and after (blue/maroon) titration with saturating quantities of unlabelled SGTA_NT. (TIF) [file pone.0113281.s003.tif]

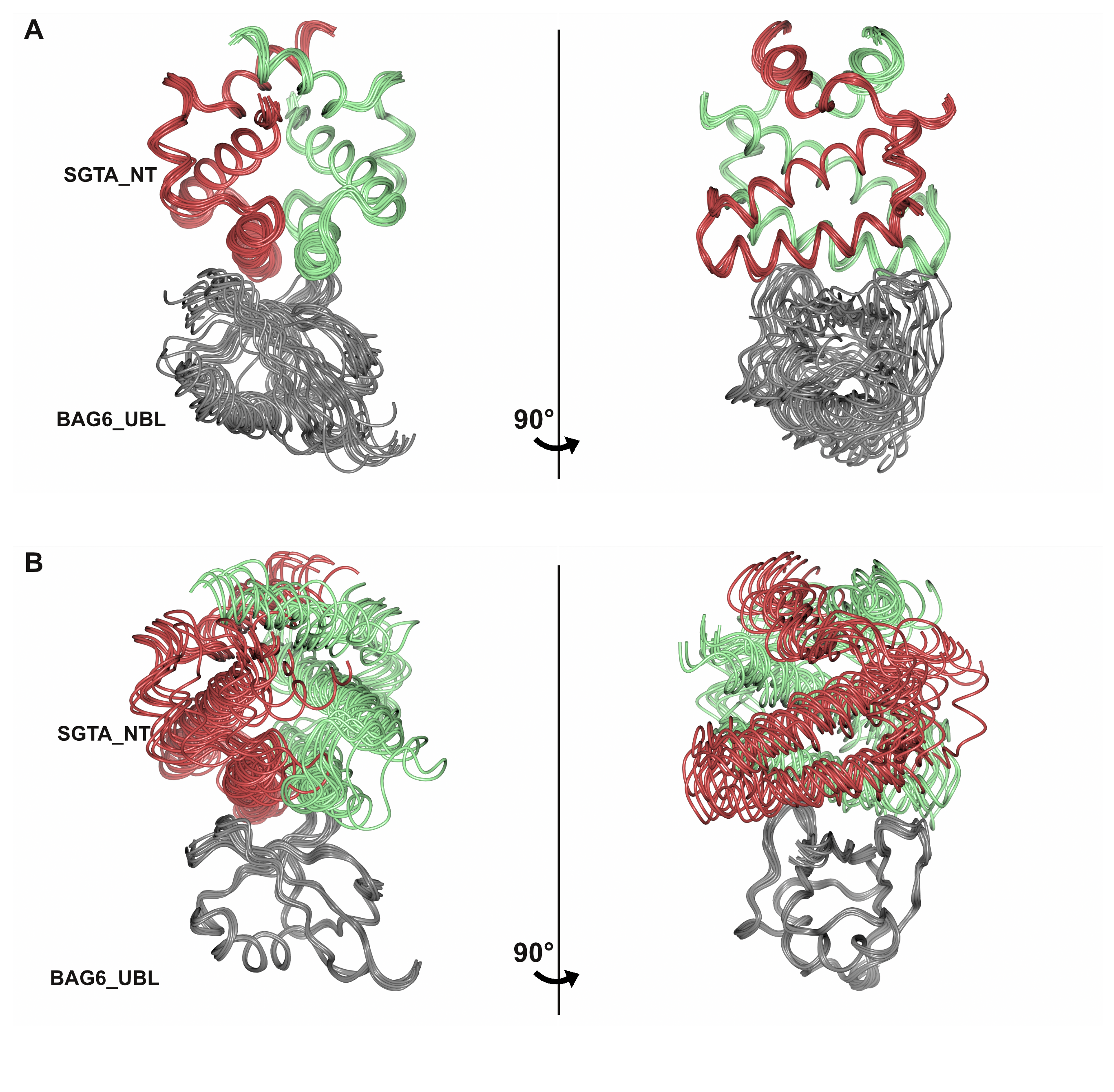

Supplement: Figure S4 — HADDOCK-generated ensembles showing 10 lowest energy structures from the top-scoring cluster for the complex between SGTA_NT and BAG6_UBL aligned according to SGTA (top) and BAG6 (bottom) structures. (TIF) [file pone.0113281.s004.tif]

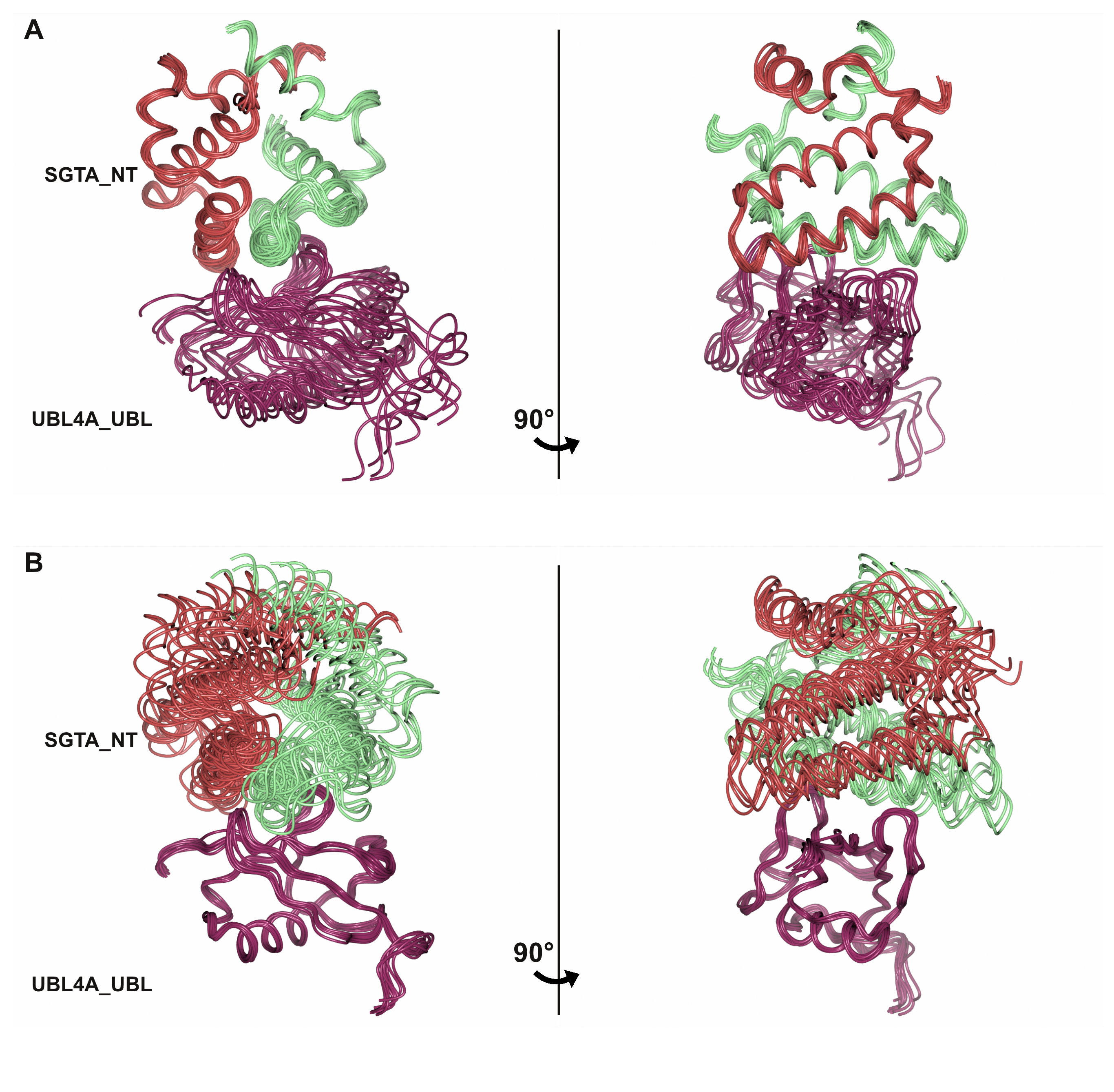

Supplement: Figure S5 — HADDOCK-generated ensembles showing 10 lowest energy structures from the top-scoring cluster for the complex between SGTA_NT and UBL4A_UBL aligned according to SGTA (top) and UBL4A (bottom) structures. (TIF) [file pone.0113281.s005.tif]

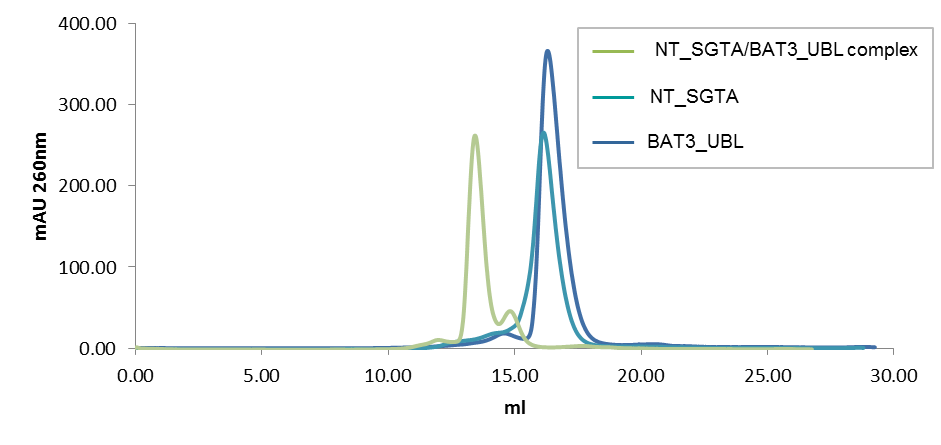

Supplement: Figure S7 — Size Exclusion Column data showing interaction between BAG6_UBL and SGTA_NT domains. (TIF) [file pone.0113281.s007.tif]
