# Supplementary material for: Solution Structure of the SGTA Dimerisation Domain and Investigation of Its Interactions with the Ubiquitin-Like Domains of BAG6 and UBL4A
Source: PLoS One. 2014 Nov 21;9(11):e113281. doi: 10.1371/journal.pone.0113281 (PMC4240585; doi:10.1371/journal.pone.0113281)
Supplement: Table S1 — Summary of Sgta_NT dimer solution structural statistics. (DOCX) [file pone.0113281.s008.docx]

|  | **Ensemble (20 structures)** |
| --- | --- |
| **Distance and Dihedral Restraints:** |  |
| NOEs: Intra-residue | 518 |
| Sequential | 345 |
| Short range | 232 |
| Medium range | 106 |
| Long range | 183 |
| Inter-monomer | 136 |
| Total unambiguous | 1520 |
| Ambiguous | 694 |
| Total NOE-derived | 2214 |
| Hydrogen bond restraints* | 68 (34 H-bonds) |
| Dihedral angles (φ + ψ)* | 126 (63 residues) |
|  |  |
| **Structural Statistics:** |  |
| Ramachandran Plot (%)** |  |
| Most favoured | 92.2 |
| Additionally allowed | 7.2 |
| Generously allowed | 0.6 |
| Disallowed | 0.0 |
| RMSD from experimental restraints |  |
| Distances (Å) | 0.027 ± 0.018 |
| Dihedrals (^o^) | 1.24 ± 1.32 |
| RMSD from idealized geometry |  |
| Bonds (Å) | 0.0034 ± 0.0007 |
| Angles (^o^) | 0.53 ± 0.101 |
| Impropers (^o^) | 1.78 ± 0.718 |
|  |  |
| NOE Violations > 0.5 Å | 0 |
| > 0.3 Å | 0 |
| > 0.1 Å | 7.7 ± 3.5 |
| **Structural Precision:** |  |
| RMSD from average structure (Å) |  |
| Backbone (all/2^nd^ary structure) | 0.95 ± 0.35 / 0.50 ± 0.16 |
| Heavyatom (all/2^nd^ary structure) | 1.35 ± 0.34 / 1.00 ± 0.15 |
|  |  |

*Applied to each monomer

**From Procheck-NMR

**Table S1:** Summary of Sgta_NT dimer solution structural statistics.
