# Supplementary material for: Solution Structure of the SGTA Dimerisation Domain and Investigation of Its Interactions with the Ubiquitin-Like Domains of BAG6 and UBL4A
Source: PLoS One. 2014 Nov 21;9(11):e113281. doi: 10.1371/journal.pone.0113281 (PMC4240585; doi:10.1371/journal.pone.0113281)
Supplement: Table S2 — HADDOCK ambiguous interaction restraints (AIRs) obtained from filtered NOE experiments for the SGTA_NT and UBL4A_UBL complex. (DOCX) [file pone.0113281.s009.docx]

| AIR | UBL4A detected residue | SGTA NOE |
| --- | --- | --- |
| 1 | L15 | S40,E44,S45,C52,L53 |
| 2 | L15 | E44,E47,Q51 |
| 3 | K55 | T55 |

**Table S2:** HADDOCK ambiguous interaction restraints (AIRs) obtained from filtered NOE experiments for the SGTA_NT and UBL4A_UBL complex.
