# Supplementary material for: Solution Structure of the SGTA Dimerisation Domain and Investigation of Its Interactions with the Ubiquitin-Like Domains of BAG6 and UBL4A
Source: PLoS One. 2014 Nov 21;9(11):e113281. doi: 10.1371/journal.pone.0113281 (PMC4240585; doi:10.1371/journal.pone.0113281)
Supplement: Table S3 — HADDOCK ambiguous interaction restraints (AIRs) obtained from filtered NOE experiments for the SGTA_NT and BAG6_UBL complex. (DOCX) [file pone.0113281.s010.docx]

| AIR | BAG6 detected residue | SGTA NOE |
| --- | --- | --- |
| 1 | V65, V81, V85 | E44,E47 |
| 2 | V65, V81, V85 | E44,E47 |
| 3 | R64,V65 | D41,E54 |
| 4 | R64,V81 | V48 |

**Table S3:** HADDOCK ambiguous interaction restraints (AIRs) obtained from filtered NOE experiments for the SGTA_NT and BAG6_UBL complex.
