# Supplementary material for: Solution Structure of the SGTA Dimerisation Domain and Investigation of Its Interactions with the Ubiquitin-Like Domains of BAG6 and UBL4A
Source: PLoS One. 2014 Nov 21;9(11):e113281. doi: 10.1371/journal.pone.0113281 (PMC4240585; doi:10.1371/journal.pone.0113281)
Supplement: File S1 — Supplemental Information. (DOCX) [file pone.0113281.s011.docx]

**Supplemental Information**

*ITC/MST Experiments on BAG6_UBL/SGTA_NT*

Our extensive efforts to study the interaction, clearly demonstrated by NMR reciprocal chemical shift perturbation, between BAG6_UBL and SGTA_NT, using ITC and MST, are outlined here. Initially, we tried the same parameters as were used for UBL4A_UBL (50mM MES pH 6, 150mM KCl) with 50µM BAG6_UBL in the cell and 500µM SGTA_NT in the syringe at 30 ºC. Analysed data indicated interaction (see Figure S6), however, fitting the isotherms using either a single-site or two-site model failed to generate a satisfactory binding curve. A set of experiments, designed to optimise the method was performed by extensively varying the following parameters alone and in tandem: buffer (20mM Tris pH 7.5, 100mM NaCl, 50mM MES pH 6, 200mM KCl and adding TWEEN at a range of concentrations between 0.04% and 0.1%); temperature (25 ºC and 30 ºC); protein concentration (adding 150-1500 µM of the sample in the syringe to the cell containing protein at concentrations between 15-150 µM and testing each protein in the cell and syringe). Unfortunately none of these attempts yielded data that could be fitted appropriately.

Similarly MST experiments at different protein concentrations were performed as well as fluorescent labelling either SGTA_NT or BAG6_UBL. A series of dilutions for unlabelled BAG6_UBL was prepared between 80 µM and 2.4 nM (or 20 µM to 0.6 nM respectively) in the presence of labelled SGTA_NT at a concentration of 0.4 or 0.2 µM. In addition, we tested the binding by using labelled BAG6_UBL at a concentration of 0.4 µM with SGTA_NT serially diluted from 400 µM to 24.4 nM. Unfortunately, no interaction between BAG6_UBL and SGTA_NT was observed in any of these experiments.
